# A Case Report of Uterine Müllerian Adenosarcoma With Sarcomatous Overgrowth

**DOI:** 10.7759/cureus.51806

**Published:** 2024-01-07

**Authors:** Mohammed Bendimya, Ouissam Al Jarroudi, Sami Aziz Brahmi, Said Afqir

**Affiliations:** 1 Medical Oncology, Faculty of Medicine and Pharmacy, Mohammed First University, Oujda, MAR; 2 Medical Oncology, Mohammed VI University Hospital Center, Oujda, MAR

**Keywords:** adjuvant chemotheapy, adjuvant radiation therapy, bilateral salpingo-oophorectomy, total abdominal hysterectomy, sarcomatous overgrowth, uterine cancer, mullerian adenosarcoma

## Abstract

Uterine adenosarcoma remains a highly aggressive tumor and is less described in the literature, with an unfavorable prognosis and an increased risk of local and distant recurrence. However, surgery, chemotherapy, and radiotherapy offer local control of the disease, and overall survival remains reduced. We report the case of a 79-year-old patient with stage IIIB uterine adenosarcoma, confirmed by immunohistochemistry and initially diagnosed with postmenopausal metrorrhagia. The patient was managed through a multimodal treatment by conducting a multidisciplinary consultation.

## Introduction

Adenosarcoma is an extremely rare gynecological tumor, the term for which was introduced by Clement and Scully in 1974 [1,2], characterized by a combination of stromal sarcoma and benign glandular component, the presence of sarcomatous overgrowth or myometrial invasion are signs of poor prognosis in this disease [3], most authors recommend total abdominal hysterectomy (TAH), usually accompanied by bilateral salpingo-oophorectomy (BSO) [4], intensive treatment with radiotherapy, chemotherapy, or endocrine therapy can be considered for high-risk patients [5,6], with the aim of improving progression-free survival (PFS) and overall survival (OS).

## Case presentation

Our patient is a 79-year-old woman referred to our department for the management of an endometrial tumor; the patient had postmenopausal metrorrhagia that had developed for more than six months and was associated with urinary burning without other digestive or urinary signs; the patient has no other pathological history.

The clinical examination did not find any abdominal or pelvic mass, and the cervix was intact with no suspicious lesions and without parametrial invasion; the patient subsequently underwent hysteroscopy with biopsy curettage, describing a histological aspect of a poorly differentiated, sarcomatoid malignant tumor process, immunohistochemistry was positive for H-Caldesmon, high molecular weight antibody (HMW- ab), smooth muscle actin (SMA), CD10 and desmin and negative for cytokeratin (AE1/AE3), cytokeratin 7, endocrine receptor (RE) and cyclin D1 consistent with uterine leiomyosarcoma.

**Figure 1:**
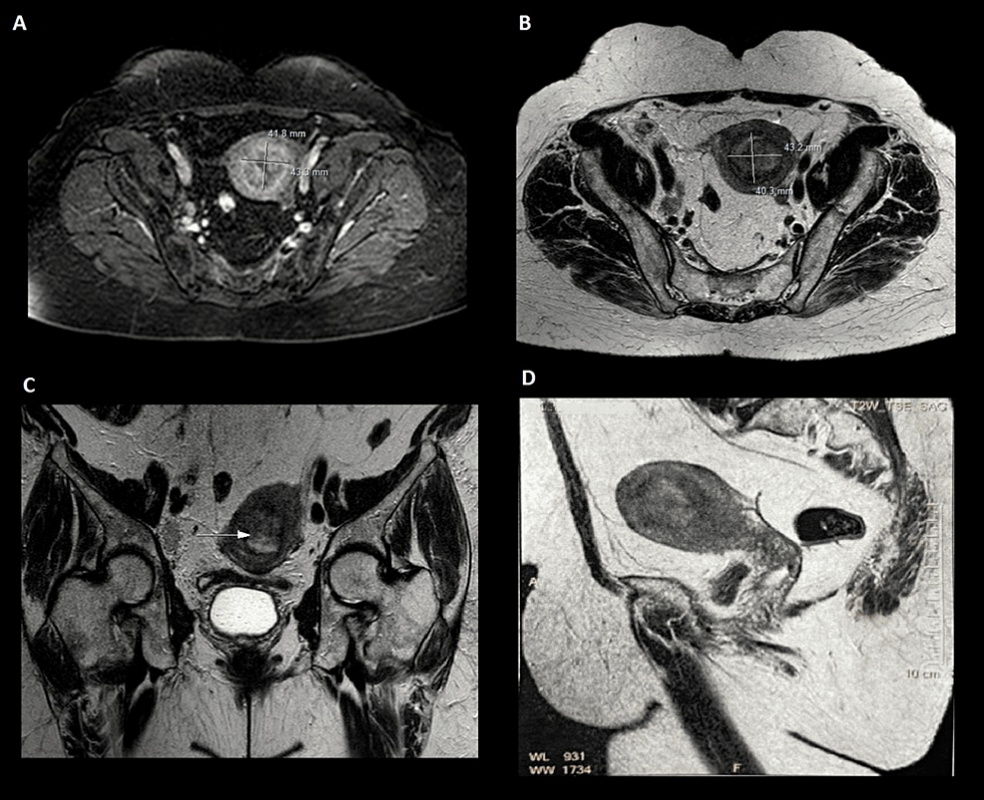
MR imaging of a 79-year-old woman with müllerian adenosarcoma of the uterus Axial T1 gado+ (A), axial T2 (B) coronal T2 (C), and sagittal T2(D) revealed endometrial tumor process (arrow), with heterogeneous hyper signal T2; after injection of contrast medium, there was moderate enhancement compared to myometrial enhancement.

A pelvic magnetic resonance imaging (MRI) revealed an endometrial tumor process, with heterogeneous T2 signal intensity, infiltrating more than half >50% of the myometrium without involvement of the serosa nor of the cervix with locoregional lymph node invasion, classified as IIIC1 according to the International Federation of Gynecology and Obstetrics (FIGO) classification (Figure 1), no visceral, bone, or lymph node metastasis has been found in the computed tomography (CT) scan.

The patient subsequently underwent a total colpo-hysterectomy, bilateral salpingo-oophorectomy, and bilateral pelvic iliac lymph node dissection.

**Figure 2:**
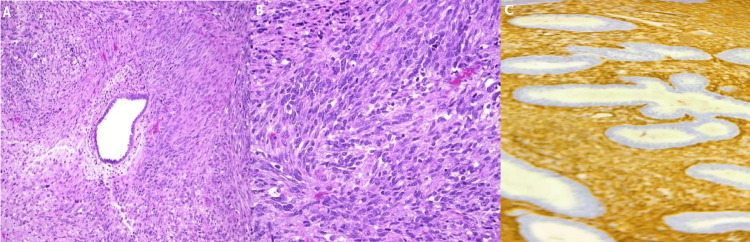
Immunohistochemical findings of the uterine mass. (A) Microphotography showing the presence of glandular and sarcomatous proliferation (hematoxylin and eosin, 200X), (B) Microphotography showing the presence of sarcomatous proliferation (hematoxylin and eosin, 200X), (C) Neoplastic cells with positive cytoplasmic expression of AML in sarcomatous part.

Anatomopathological examination revealed a biphasic tumor proliferation composed of spindle-shaped malignant stromal components that were elongated, atypical, and pleomorphic, associated with enlarged, nucleated nuclei and abundant eosinophilic cytoplasm, accounting for 80 % of the tumor, combined with the epithelial component consisting of glands, free of cytonuclear atypia, which is compressed by the sarcomatous component, forming peri-glandular cuffs, increased mitotic activity with a mitotic rate of 44 mitotic figures per high power field (HPF) along with tumor necrosis was observed, consistent with high-grade adenosarcoma (Figures 2A, 2B), the uterine isthmus and both right and left parametrium was infiltrated associated with the presence of vascular emboli, the uterine cervix, and adnexa (tube and ovary) as well as the 6 resected lymph nodes was free of tumor proliferation, reclassified as FIGO stage IlIB pT1aNOMx. Final immunohistochemistry was positive for pan-cytokeratin, estrogen and progesterone receptors, CD10, and SMA, negative for desmin and H-Caldesmon (Figure 2C).

After multidisciplinary tumor boards, treatment consisted of concomitant radio-chemotherapy followed by brachytherapy and adjuvant chemotherapy.

The patient received concurrent chemoradiotherapy with cisplatin (40 mg/m²/week) at a total dose of 48.6 Gy in 27 fractions of 1.8 Gy, followed by high-dose-rate intracavitary brachytherapy at a total dose of 10 Gy in two fractions of 5 Gy, and then 4 cycles of adjuvant chemotherapy with paclitaxel 175 mg/m² plus carboplatin (area under the curve, 5) every 21 days. During treatment, the patient developed two episodes of grade 2 neutropenia and grade 1 mucositis, which improved with symptomatic treatment; the patient had no residual neurotoxicity at the end of treatment and was placed under observation with a good follow-up.

## Discussion

Adenosarcoma is an extremely rare gynecological tumor, representing almost 9% of uterine sarcomas and 1% of female genital tract malignancies [7]. It’s an uncommon variant of mixed Mullerian tumor with a benign glandular component and a low-grade sarcomatous element; the most common localization is the uterus followed by the ovary and, in some cases, originating from the uterine cervix (2%)[1,2].

Mullerian adenosarcoma (MA) is often described in peri or postmenopausal women, with a median age of 58 years, but it can also occur in young infertile women. A few examples of extra-uterine MA have been reported [1,8,9]. In our case, we are dealing with a 75-year-old menopausal patient.

Clinical symptoms included abnormal vaginal bleeding, pelvic mass or pain, abdominal swelling, and urinary complaints, other patients experienced vaginal discharge, nausea and/or vomiting, swollen leg pain, and weight loss. [2,9-11]. Our patient presented abnormal vaginal bleeding with urinary burning.

The clinical presentation of this cancer is an enlarged uterus, polyps, or pelvic mass arising from the uterus; sometimes, the tumor protrudes into the vagina through the cervical canal. The ovary, pelvis, cervix, or peritoneum have been reported in some cases to be an extra uterine site of adenosarcoma [1,2,8-10].

On ultrasound, endometrial sarcomas can take different forms: polypoid endometrial mass, intra-myometrial mass, or even myometrial thickening [12-14].

Radiological exploration consists of preoperative pelvic diffusion MRI; due to its ability to optimize the detection of small uterine lesions, magnetic resonance images show a markedly enlarged uterus with a thin myometrium containing a polypoid mass responsible for distension of the endometrial cavity, protruding into the vagina [15]. T2-weighted images show a heterogeneous hypersignal mass with hyperintensity of the cystic areas scattered throughout. T1-weighted images show a homogeneous mass that’s iso or weakly intense relative to the myometrium with hyperintense hemorrhagic areas; the solid components of the mass show intense contrast enhancement after gadolinium administration [16,17].

Leiomyosarcomas and carcinosarcomas are the main differential diagnoses for adenosarcomas; Namimoto et al. were able to distinguish uterine sarcomas by combining two main parameters: the value of the apparent diffusion coefficient (ADC) with T2-weighted imaging [12-14,18].

Histological examination reveals a mixed tumor with a dual epithelial and sarcomatous component, characterized by cellular atypia (glandular formations as well as spindle cells) organized as hypercellular periglandular cuffs or intraglandular polypoid projections and the presence of pleomorphism and high mitotic activity. The mesenchymal component of adenosarcoma is usually a low-grade homologous sarcoma with low malignancy potential [19].

There are 2 types of adenosarcoma associated with poorer prognoses: Mullerian adenosarcoma (MA) with sarcomatous overgrowth (MASO) and MA with heterologous elements like skeletal muscle, bone or cartilage that are found in 10%-15 % of case [20].

Sarcomatous overgrowth is diagnosed when the pure sarcomatous component represents more than 25 % of the primary tumor [3]. In this case, we identified a MASO type, described as a high-grade adenosarcoma with an estimated 80% sarcomatous component.

In the case of high-grade adenosarcoma and/or sarcomatous proliferation, carcinosarcomas, leiomyosarcomas, and endometrial stromal sarcomas are the main differential diagnoses. The use of immunohistochemical markers to diagnose this rare entity is still non-specific and remains limited in this situation. Most uterine adenosarcomas share the same immunophenotype as carcinosarcomas and endometrial stromal sarcomas; they are positive for ER, PR, CD10, and WT1; these markers show lower expression when there is sarcomatous overgrowth present in uterine adenosarcoma. Other markers such as smooth muscle actin (SMA), cytokeratins, androgen receptors (AR), and desmin have variable positivity. So, it can be difficult to distinguish from the diagnoses listed above.

The genetic explanation of adenosarcomas is still poorly elucidated, and amplifications of the MDM2/CDK4 and TERT genes, abnormal activation of the PI3K/AKT, Wnt/ β-catenin pathways, and mutations of the MED12, TP53, MYLB1, APC, SMARCA4, FGFR2 and BRCA2 genes or chromosomal abnormalities have been reported [21-25].

Often, adenosarcoma is under-diagnosed and treated as a benign disease, such as an adenofibroma or polyp recurring repeatedly, and so it is difficult to make an accurate pathological diagnosis from a small histological sample, requiring further investigation of both histologically and radiologically [1,26].

Unfavorable prognostic factors include sarcomatous overgrowth, high mitotic rate, myometrial invasion, necrosis, and extra-uterine metastases [27]. Our patient combines several poor prognosis factors, including the presence of tumor necrosis, a sarcomatous proliferation estimated at 80%, and a high mitosis rate at 44 mitosis/10CFG.

The treatment of adenosarcoma requires a collective decision in multidisciplinary tumor boards, taking into account the various prognostic and recurrence factors.

According to the guidelines of the National Comprehensive Cancer Network (NCCN), the standard treatment for uterine adenosarcoma is multimodal and involves surgery, radiotherapy, chemotherapy, and hormone therapy. Surgical resection consists of a total hysterectomy with bilateral salpingo-oophorectomy [11]; evaluation of pelvic and para-aortic lymph nodes is mandatory in the presence of poor prognostic factors such as sarcomatous proliferation, myometrial invasion, or ectopic extension. Young patients desiring to preserve fertility have been treated with polypectomy alone or polypectomy with chemotherapy [5]. Additionally, bilateral salpingo-oophorectomy (BSO) is also recommended as a standard of care [19]. Total colpohysterectomy, bilateral salpingo-oophorectomy, and bilateral pelvic and iliac lymph node dissection were performed in the patient in our case.

Other risk factors may be encountered, such as lymphatic or vascular invasion. The combination of two or more risk factors with invasion of more than half the myometrium represents a high probability of recurrence and could benefit from high-dose pelvic radiotherapy with or without aggressive chemotherapy [1,2,3,6]. The NCCN recommends adjuvant radiotherapy/brachytherapy for patients with high-grade endometrial sarcomas, including adenosarcoma, to improve local disease control and reduce the risk of recurrence. Patients receive adjuvant pelvic radiotherapy using the recommended technique of external beam pelvic radiotherapy (EBRT), either alone or in combination with vaginal brachytherapy. The planned radiation dose was 50 Gy, at 1.80 Gy per fraction, in 28 fractions over five weeks [5,19,28,29].

Due to the paucity of this tumor entity, there are currently no randomized trials directly evaluating adjuvant chemotherapy. The chemotherapy agents used are those effective on mesenchymal tumors, such as the combinations of gemcitabine/docetaxel, doxorubicin/ifosfamide, doxorubicin/cisplatin, cisplatin/ifosfamide, carbo/paclitaxel, IAP (ifosfamide, epirubicin, cisplatin, mensa) and VAD (vincristine, adriamycin, dacarbazine [5,6,30], however only tumors with a sarcomatous overgrowth compound that benefits from adjuvant chemotherapy, with a 2-year survival estimated at 20% versus 100% respectively for AS+ SO and AS [31]. In our case, the patient received an adjuvant chemotherapy protocol combining intravenous paclitaxel 175 mg/m2 plus carboplatin (area under the curve- 5) every 21 days for four cycles, the courses having been well tolerated with no serious adverse effects.

The evidence of adjuvant hormonal therapy was reported for low-grade adenosarcoma without sarcomatous overgrowth expressing estrogen receptor (ER) and progesterone receptor(PR), even in recurrent and metastatic situations shown a survival improvement was reported with a median overall survival of 94 months versus 72 months in the observation group [32,33,34]. Agents used include agonists gonadotropin-releasing hormone GnRH (leuprolide), synthetic progesterones (megestrol acetate, medroxyprogesterone, dienogest), selective estrogen receptor modulators (tamoxifen, raloxifene), and aromatase inhibitors (anastrozole, letrozole) [35].

Ning et al. reported a median time to recurrence for local and distant recurrence of 46 months, with survival rates at 2 and 5 years estimated at 91.5% and 85.9%, respectively [30,31,36]. Our patient remained stable until the last follow-up, three months after the end of treatment, and showed a good performance status (PS) of 1, with no clinical or radiological signs of tumor recurrence.

## Conclusions

Adenosarcoma is a rare neoplasm of the female genital tract, occurring most commonly in the uterus. The standard of care treatment combines surgery, radiotherapy, and chemotherapy. The prognosis is considerably worsened by the presence of myometrial invasion and/or sarcomatous proliferation, which makes mullerian adenosarcoma with sarcomatous proliferation correlated with a high rate of recurrence and mortality, with most recurrences occurring in the vagina, pelvis, or abdomen, hence the need for long-term follow-up.
